# Genetic Diversity and Phylogeny of Chilli Veinal Mottle Virus in Yunnan Tobacco-Planting Regions Based on the *CP* Gene

**DOI:** 10.3390/v18080851

**Published:** 2026-08-04

**Authors:** Ning Jiang, Haonan Fang, Zhongyue Mai, Xiaotong Gai, Zhenyuan Xia, Caixiu Ya, Zuqing Hu, Dehui Xi

**Affiliations:** 1 Agronomic Research Center, Yunnan Academy of Tobacco Agricultural Sciences, Kunming 650021, China; 2 Key Laboratory of Bio-Resource and Eco-Environment of Ministry of Education, College of Life Sciences, Sichuan University, Chengdu 610064, China; 3 Southwest Bio-Resources R&D Key Laboratory of Sichuan Province, College of Life Sciences, Sichuan University, Chengdu 610064, China; 4 State Key Laboratory for Crop Stress Resistance and High-Efficiency Production, College of Plant Protection, Northwest A&F University, Yangling 712100, China

**Keywords:** ChiVMV, genetic diversity, recombination, selection pressure, phylogenetics

## Abstract

Chilli veinal mottle virus (ChiVMV, *Potyvirus capsivenamaculae*) can infect various Solanaceae crops, causing a decrease in yield and quality. In recent years, ChiVMV has become one of the most prevalent viruses affecting tobacco production, but its diversity and genetic structure are still elusive. In this study, 202 ChiVMV isolates were obtained from 13 tobacco-producing areas in Yunnan Province between 2021 and 2025. Analysis of the coat protein (CP) genes of the 202 isolates showed that there exist 193 haplotypes and abundant genetic diversity. The highest variation was observed at the N-terminus of ChiVMV CP. Further analysis displayed three high-confidence recombination events in the *CP* gene of ChiVMV Yunnan isolates, with isolate 25DLYP-7 from the Dali region playing a putative parental isolate for these recombinations. The results of neutral and selection pressure analyses indicated that the *CP* gene of ChiVMV Yunnan isolates exhibits purifying selection. Distribution analysis of these ChiVMV Yunnan isolates showed that geographical isolation is closely related to the genetic diversity of ChiVMV, and field weeds may serve as important initial sources of infection. Phylogenetic analysis demonstrated that ChiVMV Yunnan populations display close association with isolates from southwestern China, as well as isolates from South Asia and Europe.

## 1. Introduction

Chilli veinal mottle virus (ChiVMV), a member of the *Potyviridae* family and the *Potyvirus* genus, is one of the significant viruses affecting Solanaceous crops such as chili peppers, tomato, and tobacco [1]. The virus was first reported to occur on chili peppers in 1979 in the Malay Peninsula and has since spread widely across several countries and regions in Asia, including China, India, Pakistan, Indonesia, Korea, Malaysia, the Philippines, Taiwan, and Thailand, causing significant economic losses [2]. In addition, ChiVMV was also detected in Tanzania, Africa, outside Asia [3]. The virus is primarily transmitted in a non-persistent manner by various aphid species and can also spread via sap friction, with a broad host range [4]. ChiVMV was first reported in 2006 on *Capsicum chinense* in China [5]. Subsequently, it was reported to occur on *Nicotiana tabacum* and *Solanum lycopersicum* in China in 2011 and 2014, respectively [6,7]. Over the following years, it was detected in major Solanaceae crop-growing regions in China, such as Hainan, Guangxi, Sichuan, Yunnan, Chongqing, Hunan, Guangdong, and Fujian. In recent years, ChiVMV has rapidly become one of the most prevalent viruses of solanaceous crops.

The complete genome sequence of ChiVMV was first reported by Anindya in 2004 [8]. The virus has a single-stranded positive-sense genome, containing a single open reading frame (ORF) encoding a polyprotein of 3088 amino acids. This polyprotein is cleaved at nine conserved sites by proteases encoded by the virus itself, resulting in the production of ten functional proteins [8]. Chung and colleagues identified and confirmed the existence of an additional viral ORF named pretty interesting potyviridae ORF (PIPO) in the turnip mosaic virus (TuMV) genome [9]. The biological functions of potyviral proteins are both conserved and diverse [10]. Several studies have identified and cloned ChiVMV isolates from chili or tobacco in different regions of China [11,12,13,14]. Phylogenetic analysis shows that these ChiVMV isolates exhibit significant geographically driven adaptation, as well as recombination and distant transmission, demonstrating the rich diversity of ChiVMV [11,12,14,15].

The coat protein (CP) serves as a structural component of the virion of potyvirus, creating a helical filamentous coat that encapsulates the genomic RNA [16]. In addition to being a structural unit of the virus particle, the coat protein is involved in multiple biological processes, such as long-distance movement, aphid transmission, and symptom expression, playing a pivotal role in the viral lifecycle [17,18]. Analysis of the virion structure of watermelon mosaic virus (WMV) revealed that the CP protein exhibits a conserved folding pattern among all filamentous plant viruses and contains a universally conserved RNA-binding pocket. The N-terminal is exposed on the surface of the virus particle, while the C-terminal is located internally. The terminal region interacts with adjacent subunits, providing flexibility to the virus particle, suggesting that the CP protein is relatively conserved [16]. The conserved DAG motif in the N-terminal domain is necessary for aphid transmission by mediating the CP and HC-Pro interaction [10]. Thus, the functional versatility of the CP protein is a critical factor in the virus’s ability to adapt to various hosts and environmental conditions [17].

In this study, we investigated the population genetics of ChiVMV in the tobacco-planting regions of Yunnan Province, China, using the sequence of the *CP* cistron from 202 isolates collected from 13 tobacco-planting regions in Yunnan Province. We also investigated the variation, recombination, selection pressure, and phylogeny of ChiVMV Yunnan isolates. The investigation provides a new perspective for understanding the genetic diversity and evolutionary relationships of ChiVMV populations.

## 2. Materials and Methods

### 2.1. Source of ChiVMV Isolates

A total of 856 symptomatic samples were collected from 44 cities and counties in 13 tobacco-planting regions of Yunnan Province between 2021 and 2025. The principle of annual sampling is to cover the main tobacco-planting areas as much as possible and to sample as many sites as possible in each tobacco-planting area to expand the coverage of sampling. A total of 202 ChiVMV Yunnan isolates were obtained and analyzed in this study. Based on geographical origin, the 202 ChiVMV isolates can be divided into 13 populations: Yuxi (*n* = 53), Baoshan (*n* = 11), Lijiang (*n* = 19), Lincang (*n* = 20), Chuxiong (*n* = 12), Puer (*n* = 5), Dali (*n* = 13), Qujing (*n* = 8), Dehong (*n* = 2), Wenshan (*n* = 16), Honghe (*n* = 16), Kunming (*n* = 24), and Zhaotong (*n* = 3) (Table 1). The isolate names, their hosts, and sites of collection are shown in Supplementary Table S1. ChiVMV isolates from other regions and other hosts used for constructing a phylogenetic tree are shown in Supplementary Table S2.

### 2.2. RNA Extraction, RT-PCR Amplification, and Sequencing

Total RNA was extracted from 0.1 g of the sample using the TRIzol reagent (ProbeGene, Xuzhou, China) and then treated with DNase (Sangon, Shanghai, China) [19]. The quality, purity, and concentration of the extracted sample RNA were measured using the NanoDrop One spectrophotometer (Thermo Fisher, Waltham, MA, USA). cDNA for PCR was synthesized using the M-MLV Reverse Transcriptase kit (Invitrogen, Carlsbad, CA, USA) and stored at −20 °C. Specific amplification primers, ChiVMV-CP-F: 5′-GCAGGAGAGAGTGTTGATGC-3′ and ChiVMV-CP-R: 5′-TCATAATCCCCGAACGCCTAG-3′, were designed based on the *CP* sequence of the ChiVMV isolate Yp8 (KC711055.1) [13]. Specific sequencing primers, ChiVMV-CXCP-F: 5′-AAGTAACACAAGGGCATTGC-3′ and ChiVMV-CXCP-R: 5′-CGCTTCAGCAAGATTGCTAA-3′, were designed based on the *CP* sequence of ChiVMV isolate Yp8 for bidirectional sequencing. The obtained sequences that meet quality standards were assembled using SnapGene 5.2.

### 2.3. Sequence Diversity, Geography, and Host Adaption Analysis

The classical MDS was used to compare the *CP* gene sequences of the 202 ChiVMV Yunnan isolates, and the nucleotide and amino acid sequence identities were calculated [20]. Highly variable regions are identified based on the original per-site entropy and SNP frequency. Sites with the top 4% of original entropy and SNP frequency ≥ 0.15 are defined as seed sites, and seed intervals within ≤17 bp are merged into continuous regions. Subsequently, regions with length ≥ 10 bp, ≥2 seed sites, seed density ≥ 0.08, and average original entropy ≥ 0.17 are retained. The sample order is determined by the first axis of classical MDS from the pairwise nucleotide distance matrix.

The correlation between geographic distance and genetic distance was tested by the Mantel test. The statistic is the Pearson correlation coefficient between the upper triangular elements of two distance matrices, with 999 one-sided permutations performed [21]. The explanatory power of host origin on genetic distance structure was evaluated using univariate PERMANOVA. The analysis calculates pseudo-F and R^2^ based on the p-distance matrix of CP sequences and performs 999 label permutations [22]. Subsequently, the PERMDISP test was performed to calculate the distance from the sample to the centroid of the group in the PCoA space that contains both positive and negative characteristic axes [23]. Sequence processing and visualization were accomplished using custom Python scripts. The custom process runs on Python 3.14.4, mainly using NumPy 2.4.3, pandas 3.0.3, matplotlib 3.10.9, and Biopython 1.87. The script is used for input checking, metadata matching, distance matrix construction, standard statistic calculation, result summarization, and graphic drawing.

### 2.4. Analysis of Variable Sites and Conserved Regions

DnaSP 6.12 was used to analyze the haplotypes, variations, and conserved regions of the *CP* gene of the 202 ChiVMV Yunnan isolates [24]. Haplotypes were inferred by collapsing identical sequences, and haplotype diversity (Hd) was calculated to quantify genetic complexity within the population. To identify conserved blocks, a sliding-window analysis of nucleotide diversity was performed with a window length of 100 bp and a step size of 25 bp. Genomic regions exhibiting significantly reduced π values compared to the genome-wide average were designated as conserved regions. This combined approach of haplotype profiling and diversity scanning allowed for a robust delimitation of hypervariable sites and structurally constrained conserved domains within the ChiVMV *CP* gene.

### 2.5. Recombination Analysis

Recombination sites were identified using seven detection programs in RDP 4.101: R (RDP), G (GeneConv), B (Bootscan), M (MaxChi), C (Chimaera), S (SiScan), and 3S (3SEQ). Only those recombination events predicted by at least five of the abovementioned methods and with a *p* value < 0.05 were considered as valid [25].

### 2.6. Population Genetics Analyses

The haplotype diversity (*H*_d_) and nucleotide diversity (*P*_i_) of the ChiVMV population in Yunnan tobacco-planting areas were calculated using DnaSP 6.12. Tajima’s D and Fu’s *Fs* statistics for the ChiVMV Yunnan population were calculated using Arlequin 3.5 to perform neutral analysis. Significance was evaluated by performing 1000 resampling tests [24].

### 2.7. Selection Pressure Analysis

The *d*N (non-synonymous mutation rate) and *d*S (synonymous mutation rate) at each site of the ChiVMV *CP* gene were calculated using the Nei–Gojobori method with Jukes–Cantor correction in MEGA X. The *d*N/*d*S ratio (ω) was used to evaluate the selection pressure on the *CP* gene of each population: ω < 1 suggests purifying selection (negative selection), ω = 1 indicates neutral selection, and ω > 1 implies positive selection (diversifying selection) [26].

### 2.8. Phylogenetic Analyses

A total of 26 sequences of ChiVMV *CP* genes, domestic and international, from the NCBI database (https://www.ncbi.nlm.nih.gov/) (Supplementary Table S2) and 26 sequences from the present study (Supplementary Table S4) were used to construct the phylogenetic tree. MAFFT v7.526 was used to perform multiple sequence alignment [27]. The optimal substitution model was automatically selected by ModelFinder using IQ-TREE V3.1.1 to construct a maximum likelihood tree [28]. The tree was then subjected to 1000 ultrafast bootstrap replicates and 1000 SH-aLRT tests to evaluate node support rates. High support nodes are defined as UFBoot ≥ 95 and SH aLRT ≥ 80. The treefile output by IQ-TREE was used for subsequent visualization. Graphic drawing was completed using custom Python scripts.

## 3. Results

### 3.1. Sequence Analysis of Coat Protein Gene of ChiVMV Yunnan Isolates

**Figure 1:**
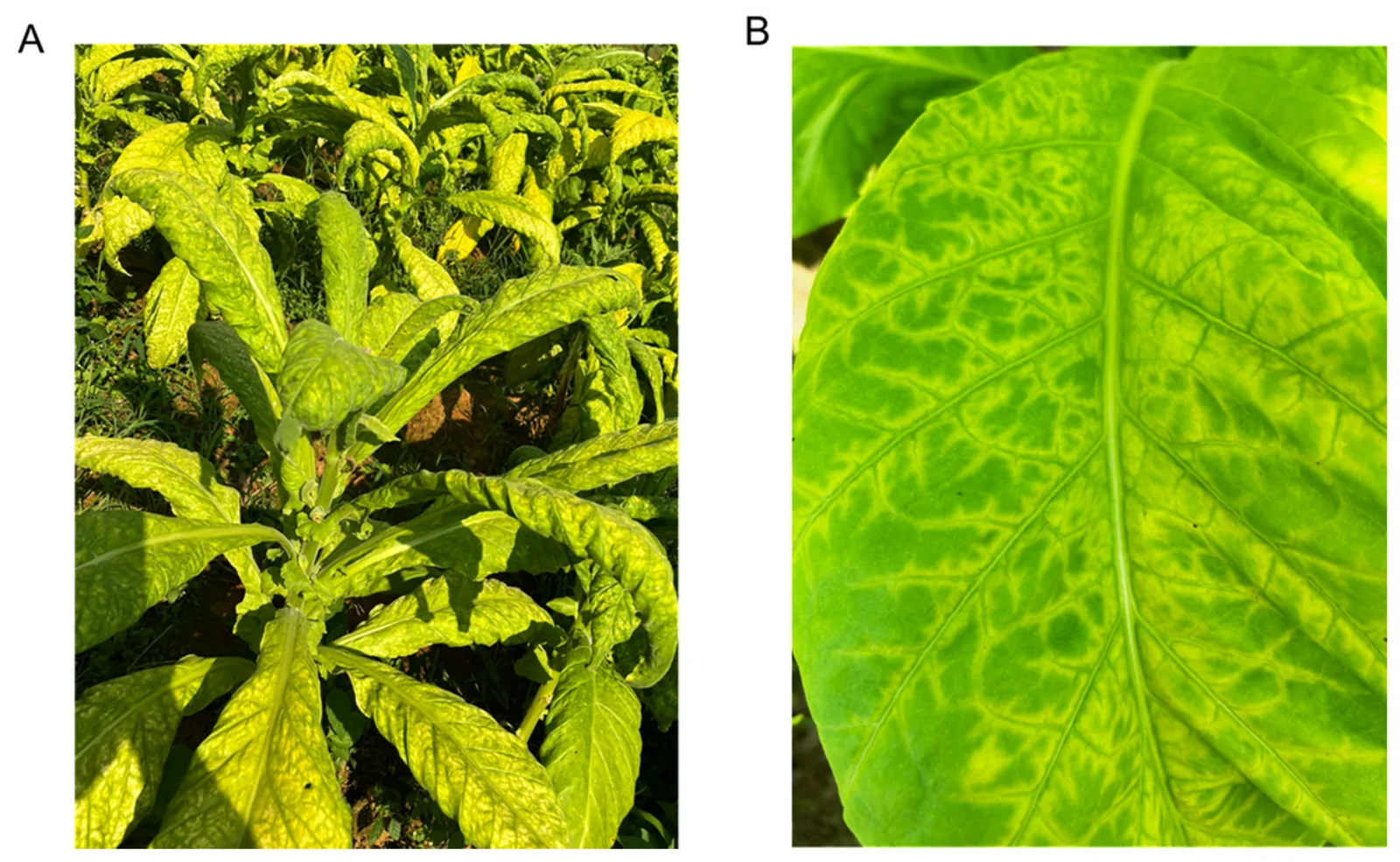
Typical symptoms of ChiVMV on field-grown tobacco plants collected from Lijiang city. (**A**) Leaf curling symptom. (**B**) Chlorosis bands along the leaf veins.

To investigate the occurrence and diversity of ChiVMV in Yunnan tobacco-planting areas, a total of 202 ChiVMV isolates were obtained from 856 samples collected from 44 cities and counties in 13 tobacco-planting regions in Yunnan Province. The typical symptoms of ChiVMV on tobacco grown in the field are leaf curling and chlorosis bands along the leaf veins (Figure 1). All samples were tested for infection with ChiVMV using a specific pair of primers that amplify the *CP* cistron. RT-PCR and sequencing yielded *CP* gene sequences of 202 ChiVMV isolates, with 172 isolates obtained from tobacco and 30 isolates from vegetables or weeds surrounding tobacco fields, including *Solanum lycopersicum*, *Capsicum annuum*, *Solanum tuberosum*, *Solanum nigrum*, and *Solanum muricatum* from the *Solanaceae* family, *Bidens pilosa* and *Laggeraalata(Roxb.)Sch.-Bip.* from the *Asteraceae* family, and *Clerodendrum bungei* from the *Lamiaceae* family. The GenBank accession numbers of the sequences obtained in the present study are PZ717641–PZ717842 (Supplementary Table S1).

**Figure 2:**
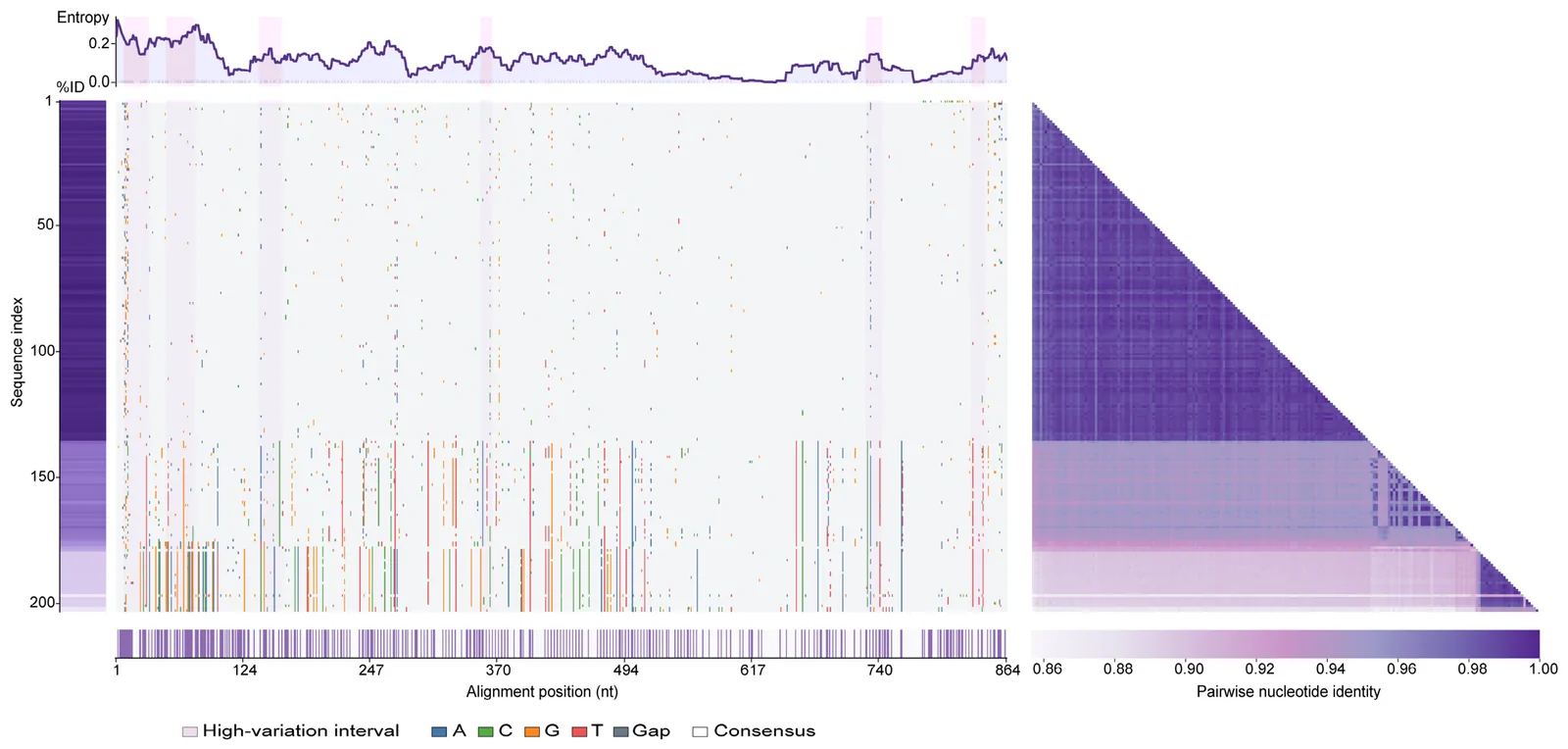
Diversity and nucleotide sequence consistency of the *CP* gene of ChiVMV Yunnan isolates. The top curve represents the smooth Shannon entropy calculated along the *CP* gene alignment position, which is used to reflect the intensity of variation at different loci. The light purple stripes indicate the high variability range. The middle matrix displays the base variation status of each sequence relative to the consensus sequence, with light gray indicating consistency with the consensus sequence and colored vertical lines representing A, C, G, and T states, respectively. The left color band represents the nucleotide consistency between each sequence and the consensus sequence. The inverted triangle heatmap on the right represents the nucleotide consistency between 203 sequences. All sequences are sorted according to MDS based on nucleotide distance, so that similar sequences are arranged adjacent to each other in the graph.

Multiple sequence alignment of the full-length *CP* gene sequences of 202 ChiVMV Yunnan isolates was performed using classical MDS. The result showed that the nucleotide sequence identity among isolates from different geographical regions ranged from 85.6% to 100%. It is worth noting that the ChiVMV *CP* gene showed significant variation at the 5′ end (Figure 2). The identity of the deduced amino acid sequence of ChiVMV CP ranged from 91.2% to 100%, with the highest degree of variation at the N-terminus (Supplementary Figure S1).

### 3.2. Variation, Conservation, and Recombination Analysis on CP Gene of ChiVMV Yunnan Isolates

It was reported that the variation and recombination often occur in the potyvirus genome [10]. Haplotype analysis of the 202 ChiVMV *CP* gene sequences was performed using DnaSP 6.12. The result displayed that there exists a high degree of haplotype diversity in the ChiVMV isolates from Yunnan tobacco-planting areas. In the present study, 193 unique haplotypes were identified, with a haplotype diversity index (*H*_d_) of 0.9994, indicating extremely high genetic polymorphism in ChiVMV across tobacco-growing areas in Yunnan Province. No dominant haplotype was observed in different populations: 176 haplotypes consisted of a single sequence, with only two haplotypes containing multiple sequences—Hap_14 (*n* = 3, from Chuxiong) and Hap_122 (*n* = 3, from Qujing) (Supplementary Table S3).

Next, mutation analysis was performed. The result revealed that the average G+C content of the 202 ChiVMV *CP* gene was 43.7%. A total of 319 variable sites were identified, accounting for 37.05% of all sites, with 228 (71.5%) being simplified information sites and 91 single-nucleotide variant sites (Table 2). No insertions or deletions were detected. The above results indicate that the ChiVMV *CP* gene has a stable structure in the ChiVMV Yunnan population, with nucleotide substitution being the only form of variation.

We then performed the conservation analysis of the ChiVMV *CP* gene sequence using DnaSP 6.12. The result revealed that the sequence conservation of ChiVMV in Yunnan tobacco-planting areas reached 63.0% (C = 0.630), with preservation values of 0.728 and 0.747 in two conserved regions (Table 3), indicating that the *CP* gene in ChiVMV Yunnan isolates is relatively conserved.

Finally, the recombination events among ChiVMV Yunnan isolates were detected. We identified three high-confidence recombination events in the *CP* gene of the 202 ChiVMV isolates (Table 4). 25DLYP-7 (PZ717751), an isolate from the Dali region, was identified as a putative parental sequence in three inferred recombination events, appearing as the major parent in two recombinants and as the minor parent in another recombinant.

### 3.3. Phylogenetic Analysis of ChiVMV Yunnan Isolates

**Figure 3:**
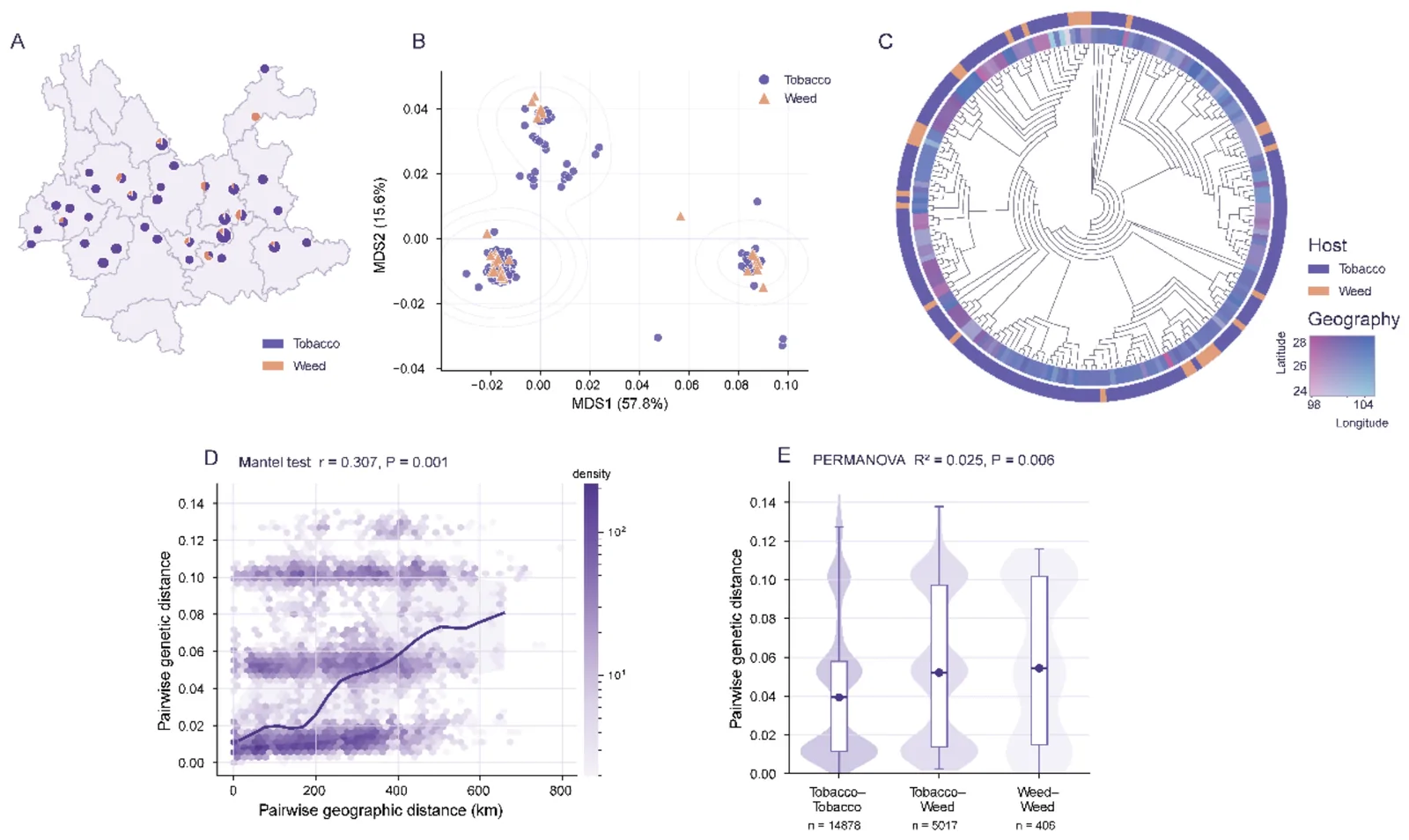
Geographical structure and host origin relationship of *CP* gene sequence differentiation of ChiVMV Yunnan isolates. (**A**) Distribution map of sampling locations in Yunnan Province, with round cakes representing merged sampling points and sector colors representing the proportion of tobacco and weed sources. (**B**) MDS dimensionality reduction map based on pairwise genetic distance of *CP* genes, displaying the distribution of tobacco and weed source samples in sequence space. (**C**) A phylogenetic tree constructed based on the maximum likelihood method, where the inner circle represents geographic origin and the outer circle represents host origin. (**D**) The relationship between the geographical distance and genetic distance of each pair of samples, with the color depth indicating the density of the sample pairs. (**E**) The distribution of paired samples’ genetic distance between different host combinations.

The occurrence, evolution, and variation of plant viruses are influenced by factors such as host species, geographical location, and growth environment [29]. To investigate the impact of geographical and host factors on the evolution of ChiVMV Yunnan population, the geographical structure and host origin relationship of *CP* gene sequence differentiation of ChiVMV Yunnan isolates were analyzed (Figure 3A). The MDS dimensionality reduction map showed that distribution of tobacco and weed source samples displays a staggered distribution in the main clusters (Figure 3B). The weed source samples are distributed in multiple phylogenetic branches, indicating a lack of obvious host source aggregation in *CP* gene sequence differentiation (Figure 3C). The Mantel test showed a moderate positive correlation between geographic distance and genetic distance for each pair of samples (*r* = 0.307, *p* = 0.001), indicating that sequence differentiation has a certain spatial geographic structure (Figure 3D). As shown in the box plot in Figure 3E, there is some overlap in the distribution of the three types of combinations. PERMANOVA analysis exhibited some overlap in the distribution of different host combinations (*R*^2^ = 0.025, *p* = 0.006), indicating that although statistically significant, the host source can only explain about 2.5% of the genetic distance differences in *CP* genes. The above results indicate a moderate association between genetic and geographic distance, with weak influence from host sources. In addition, we conducted a comparative analysis of the genetic structure of *CP* sequences from ChiVMV isolates obtained in different years in Yunnan tobacco-growing regions. The results showed that although ChiVMV isolates from the same year may exhibit relative clustering due to similar geographical sampling sources, there is no clear genetic structure association of *CP* sequences among samples collected in different years (Supplementary Figure S2).

### 3.4. Genetic Diversity and Selection Pressure of ChiVMV Yunnan Isolates

It is known that there is a close relationship between selection pressure and genetic diversity within virus populations [30]. Genetic diversity parameters of 13 ChiVMV populations from Yunnan tobacco-planting regions were calculated using DnaSP 6.12. The results revealed that the genetic diversity of the ChiVMV population in Yunnan tobacco-growing areas is relatively high, with a haplotype frequency of up to 95.1% (Table 5). There were significant differences in genetic diversity levels among different regions. The nucleotide diversity was higher in areas such as Dehong and Baoshan, while it was lower in regions such as Qujing and Honghe.

Neutral analysis was then performed using Arlequin 3.5. The result showed that Tajima’s D and Fu’s *Fs* were significantly negative in the isolates from Yuxi, and Fu’s *Fs* was significantly negative in the isolates from Honghe, Kunming, and Lijiang, indicating that the ChiVMV populations in these regions may have recently experienced population expansion events. However, very few isolates (e.g., *n* = 2–3) may limit the robustness of neutrality tests and diversity estimates. In other regions, neutrality test statistics were not significant, suggesting that ChiVMV populations in these regions are in a relatively stable evolutionary state, consistent with the neutral evolution model (Table 5).

Research has shown that the type of selective pressures (positive selection, purifying selection, or neutral selection) acting on the *CP* gene during evolution directly mirrors the virus’s adaptive evolutionary strategy [15]. The non-synonymous (*d*N) and synonymous (*d*S) mutation rates of the *CP* gene for the ChiVMV populations were calculated using MEGA X. The results revealed that the *d*N/*d*S values for the 13 ChiVMV populations from tobacco-growing regions were all less than 1 (ranging from 0.039 to 0.129), indicating that most mutations in these populations were synonymous. This indicates that the mutations in these 13 ChiVMV populations are predominantly synonymous, and the evolution of the *CP* gene in the ChiVMV Yunnan population may be mainly driven by purifying (negative) selection pressure (Table 6).

### 3.5. Association Analysis of ChiVMV Isolates from Weed or Tobacco in Different Seasons

**Figure 4:**
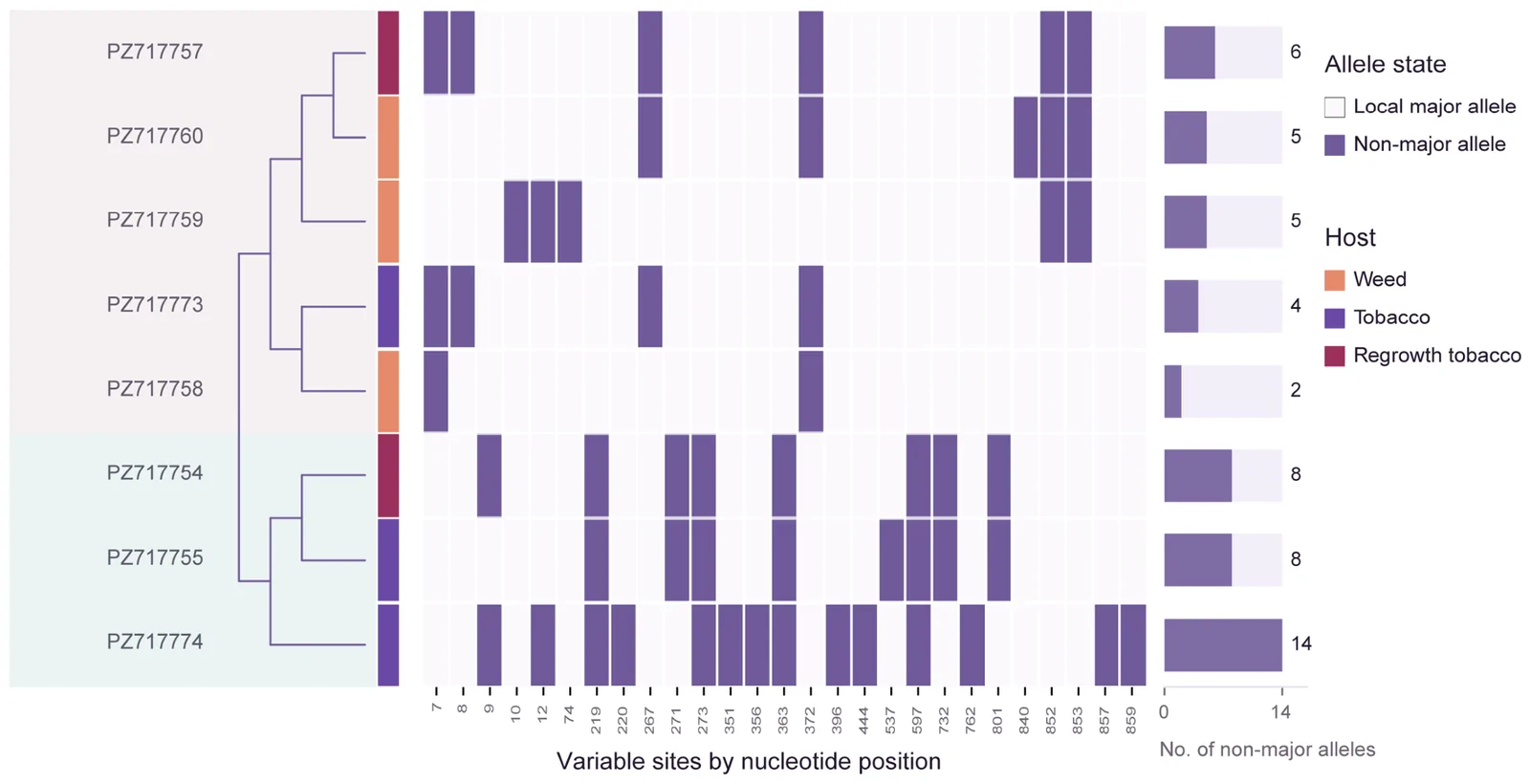
Variation structure of the ChiVMV *CP* gene in the Lijiang local cluster. The heatmap displays the allelic status of local samples in Lijiang at the *CP* gene mutation site. The BIONJ distance tree was generated using IQ-TREE V3.1.1 under GTR + F + I settings. The column represents the variant sites arranged by *CP* position. The purple square represents the variation of the sample at the corresponding site relative to the local cluster master allele state. The color bar on the left indicates the source of the host, while the bar graph on the right represents the number of *CP* differential sites for each sample relative to the main allele state.

Previous studies have shown that weeds in the field play an important role in the spread of diseases [25]. In the present study, the association of isolates from weeds, spring tobacco, summer tobacco, and regrowth tobacco growing naturally in the field of last cultivation season was analyzed. The results showed that the *CP* sequence of 25HPWLYY121-1 (accession number PZ717755) from Wenle spring tobacco was highly similar to the *CP* sequence of 25HPWLCCY-1 (accession number PZ717754) from the tomato field around the spring tobacco field. Similarly, the *CP* sequence of 25HPXZCCY-1 (accession number PZ717757) from Huaping regrowth tobacco was almost identical to the *CP* sequence of 25HPXZSn-5 (accession number PZ717760) from *Solanum nigrum* around the field. In the meantime, 25HPXZCCY-1 (accession number PZ717757) and 25LJHP-11 (accession number PZ717773) from tobacco and 25HPXZSn-5 (accession number PZ717760), 25HPXZSn-2 (accession number PZ717758), and 25HPXZSn-4 (accession number PZ717759) from *Solanum nigrum* clustered into one main clade (Figure 4). The above results indicated that ChiVMV on weeds and regrowth tobaccos may serve as the initial sources of infection for tobacco planted in the next cultivation season.

### 3.6. Phylogenetic Analysis of Yunnan, Domestic and International ChiVMV Isolates

It has been reported that the spatiotemporal dynamics and evolutionary history of ToLCPalV are related to its spread in South and Western Asia [31]. To investigate the phylogeography and evolutionary dynamics of ChiVMV Yunnan isolates from tobacco-planting regions, a total of 52 sequences of ChiVMV *CP*, including 8 sequences from other countries, 18 sequences from different provinces and different hosts in China, and 26 sequences from the present study, were used to construct a phylogenetic tree.

The results indicated that isolates from the central and eastern regions of Yunnan Province clustered primarily in one branch, while those from the southwestern region of Yunnan Province exhibited multiple/diverse clustering. For isolates from other provinces in China, the isolates from Guizhou (OP378160.1) and Sichuan (KC711055.1, PX687011.1, MK405594.1) in southwestern China are clustered together with most of the isolates from Yunnan, while the isolates from Hainan (GQ981316.1), Jiangsu (PP582383.1), Hunan (KR296797.1), Guangdong (KU987835.1), and Guangxi (MT782116.1) are clustered together. It is worth noting that the isolate 25DLYP-7 (PZ717751) from Dali, Yunnan, and the isolate (MG674071.1) from Liaoning, Northeast China, cluster into an evolutionary branch. The above results indicate that the phylogenetic relationship between ChiVMV isolates from Yunnan tobacco-planting regions and those from southwestern regions of China is relatively close, but there is also a long-distance phylogenetic association in terms of geographical distance.

**Figure 5:**
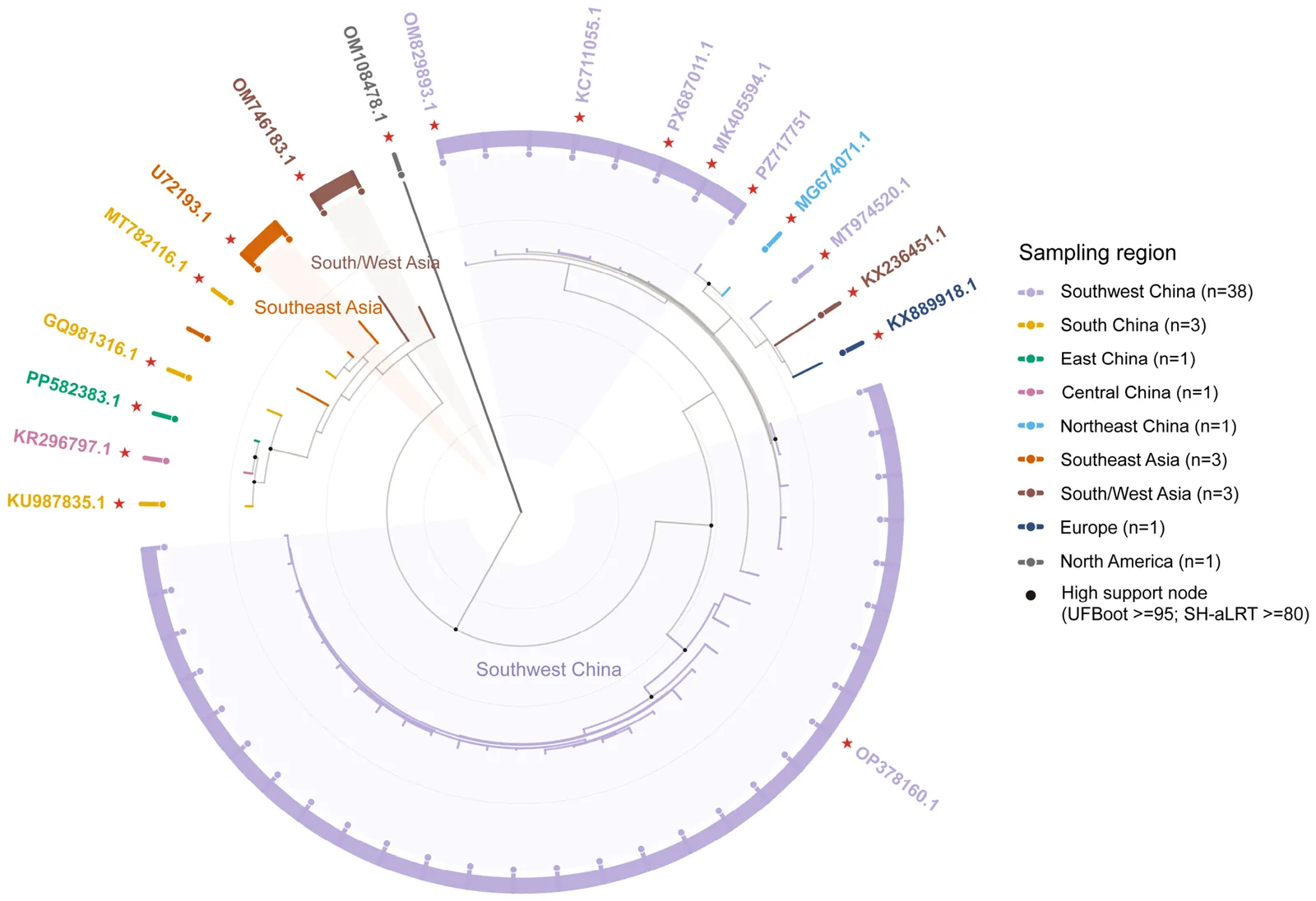
Phylogenetic analysis of Yunnan domestic and international ChiVMV isolates. The tree was constructed using IQ-TREE V3.1.1 to automatically select the optimal replacement model based on ModelFinder. The treefile output by IQ-TREE is used for subsequent visualization. The circular tree uses midpoint rooting during the drawing phase, with the midpoint of the longest leaf path as the display root, to unify the graphic layout and branch arrangement. Phylogenetic interpretation is based solely on the topological clustering relationships and node support between samples. Samples from different regions are coded with different colors. The highlighted samples are marked with asterisks and GenBank accession numbers. To improve the identification of short branches in circular graphs, only the cumulative branch length from root to node is transformed radially in the drawing coordinates by (branch length/max depth) ^0.58.

From an international perspective, the isolate 25DLYP-7 (PZ717751) from Dali, Yunnan, is grouped with ChiVMV pepper isolates from Pakistan (KX236451.1) and Italy (KX889918.1), indicating that certain strains from Europe and South Asia are closely related to 25DLYP-7. The ChiVMV isolates from Asia, including Saudi Arabia (OM746183.1), India (MK165661.1), Thailand (U72193.1), Vietnam (DQ925440.1), and Indonesia (AB703255.1), have a closer evolutionary relationship with isolates from Hainan, Guangdong, and other regions of China. And the ChiVMV *Basella alba* isolate from the United States (OM108478.1) can almost serve as an outgroup, possibly representing an ancient subtype or distant isolate of ChiVMV. The above highlighted isolates are marked with asterisks and GenBank accession numbers in the phylogenetic tree(Figure 5).

## 4. Discussion

The potyviruses are the largest genus in *Potyvirids* and include more than 150 distinct viruses found worldwide [18]. This study systematically analyzed the genetic diversity, population structure, and evolutionary characteristics of ChiVMV populations, based on the *CP* gene sequences of 202 ChiVMV isolates from 13 major tobacco-planting regions of Yunnan Province. Compared with soybean mosaic virus (SMV), which belongs to the same genus of *Potyvirus*, ChiVMV Yunnan isolates possess a relatively higher nucleotide diversity (0.046) than SMV (0.020) [32]. This may be related to its aphid transmission mode and wider host range, reflecting the differences in diversity strategies among different viruses. Mutation and haplotype diversity analysis indicate that the ChiVMV population in Yunnan tobacco-growing areas has a high degree of genetic polymorphism.

Although there exists a high level of diversity in the *CP* gene of ChiVMV Yunnan isolates, there are also two highly conserved regions (nt 376–489 and nt 519–791). The CP core domain is conserved, while there is a higher degree of variation at the N-terminus. This finding is consistent with the analysis of the structure of WMV viral particles, which indicated that the N-terminus of the CP protein is exposed on the surface of the viral particle. The terminal regions interact with adjacent subunits and contribute to the flexibility of the viral particle. This structural feature suggests that the N-terminal region may be subject to different selective pressures [16]. Further studies on the CP protein of potato virus Y (PVY) confirmed that both the N- and C-terminal regions of the CP protein have structural plasticity, allowing them to perform various biological functions at different stages of the viral life cycle [17]. In the present study, the higher variation observed in the N-terminal region of the ChiVMV *CP* may contribute to their adaptation and is likely related to interactions between the virus and host factors, aphid transmission efficiency, and immune evasion.

The occurrence of recombination events may accelerate viral adaptive evolution, facilitate the formation and spread of new viral strains, and pose a potential challenge to virus control [33]. In the present study, we identified three high-confidence recombination events in the ChiVMV Yunnan population, with the Dali isolate 25DLYP-7 serving as a parental strain involved in the recombination events. This finding is similar to the study by Seo et al. on SMV, which identified 19 clear recombination events among 44 SMV isolates, with approximately 64% of the isolates confirmed as recombinants [32]. A recent study on WMV revealed that recombination is common within viral genomes and that the CP region can form recombination hotspots [34]. Dali is located on the western border of Yunnan Province, and human activities and exchanges may promote new interactions between plants and viruses, with recombination playing a key role in these new interactions.

Selection pressure analysis revealed that the *d*N/*d*S values for the 13 ChiVMV populations from tobacco-planting regions were all less than 1, indicating that most mutations in these populations were synonymous. This suggests that the phylogeny of the *CP* gene in the ChiVMV Yunnan population is primarily driven by purifying selection. This result aligns with the classical discussion on plant virus population genetics, which states that negative selection leading to reduced genetic diversity is a common phenomenon among plant viruses, though it may depend on the dynamic interactions between the virus and its host plant population [30]. Compared to other viruses in the potyviruses, the purifying selection pressure on the ChiVMV *CP* gene is moderate. The overall *d*N/*d*S value of ChiVMV Yunnan isolates was 0.051, similar to the *d*N/*d*S value of the SMV *CP* gene (0.033) [32] but lower than the reported values for the *CP* gene of PVY [26,35].

The complex terrain and climatic gradients, diverse planting systems, frequent agricultural practices, and seasonal migration of aphid populations in Yunnan together create favorable conditions for the transmission and spread of plant viruses. Population genetic structure analysis revealed that the nucleotide diversity of ChiVMV isolates was higher in border regions such as Dehong and Baoshan, suggesting a potential risk of foreign virus introduction or local diversification. Previous studies have reported that weeds in greenhouses and tobacco fields are important overwintering hosts for tomato wilt virus (TSWV). In tobacco-planting areas, weeds are not only the host of overwintering vector insect thrips, but the pathogens carried by thrips are also the key source of epidemic outbreaks [36]. It was also found that non-crop hosts play key roles in the persistence and spread of raspberry bushy dwarf virus (RBDV) [37]. In addition, Sattar et al. found that the isolates of tomato leaf curl Palampur virus (ToLCPalV) displayed clear geographic clustering but did not exhibit host-specific differentiation [31]. In the present study, the 30 isolates obtained from weeds clustered together with tobacco isolates. There is a high degree of sequence consistency between tobacco and weeds growing in nearby fields, suggesting that field weeds may also serve as potential virus reservoirs and infection sources for ChiVMV, and the genetic differentiation of ChiVMV Yunnan isolates is primarily associated with geographic isolation rather than host adaptation. Another study showed that the population genetics of alfalfa mosaic virus (AMV) in China displayed significant differences in viral populations across different bioclimatic zones [25]. Thus, appropriate agricultural management practices involving removal of weeds may be important for ChiVMV control.

## 5. Conclusions

In summary, the ChiVMV Yunnan population demonstrates relatively high genetic diversity and a complex population structure, with geographic isolation being closely related to their genetic differentiation. Although recombination events and local amplification jointly shape the rich variability of the ChiVMV Yunnan isolates, the *CP* gene exhibits purifying selection. High variability exists in its surface-exposed regions, potentially allowing the virus to adapt to different hosts or vectors, while a conserved core domain may maintain its function of binding to vRNA and forming a virion. Phylogeny analysis revealed a polymorphic pattern among ChiVMV Yunnan isolates, which has a close phylogenetic association with isolates from southwestern China, as well as isolates from South Asia and Europe, displaying cross-border dispersal patterns of ChiVMV Yunnan isolates. However, there exists limitation of population genetic and phylogenetic inferences on a single genomic region (the *CP* gene). Therefore, future work requires more research on virus epidemics, transmission vectors, and pathogenic factors. Developing disease-resistant crops, integrating weed removal, controlling vector insects, and strengthening international plant quarantine cooperation are expected to alleviate the further spread of ChiVMV.

## Figures and Tables

**Table 1:** The number of ChiVMV isolates obtained from 13 tobacco-planting regions in Yunnan Province.

Region	Number of ChiVMV Isolates	Region	Number of ChiVMV Isolates
Baoshan	11	Lijiang	19
Zhaotong	3	Lincang	20
Chuxiong	12	Puer	5
Dali	13	Qujing	8
Dehong	2	Wenshan	16
Honghe	16	Yuxi	53
Kunming	24		

**Table 2:** Variation analysis of the *CP* gene in 202 ChiVMV Yunnan isolates.

Number of Sequences	Mean Percentage of G + C	Number of Variable Sites			Mutation Type	
		Parsimony Informative Sites	Singleton Variable Sites	Total Number of Variable Sites	Insertion	Deletion
202	43.7	228	91	319	0	0

**Table 3:** Conserved loci of the *CP* gene in 202 ChiVMV Yunnan isolates.

Conserved Loci	Start-End Site/nt	Conservation	Consistency	*p* Value
1	376–489	0.728	0.944	0.0116
2	519–791	0.747	0.978	<0.0001

**Table 4:** Recombination analysis of *CP* gene in 202 ChiVMV Yunnan isolates.

Recombinant	Major Parent	Minor Parent	Detection Methods						
			R	G	B	M	C	S	T
23LC-1	25DLYP-7	25CXYR-15	+	−	+	+	+	+	+
25BSTC-14	25DLYP-7	22BSLJ4-1	+	+	+	+	+	+	+
23WSQBG3-3	25HPWLYY301-1	25DLYP-7	+	+	+	+	−	+	+

“+” indicates that the method detected a *p *< 0.05, “−” indicates that the method did not detect a *p* < 0.05.

**Table 5:** Genetic diversity analysis of *CP* genes in 202 ChiVMV Yunnan isolates.

Population	Tajima’s D	Fu’s *F*s	Haplotype Diversity (*H*_d_)	Nucleotide Diversity (*P*_i_)	Number of Haplotypes (h)
Baoshan	0.669 ^ns^	−0.212 ^ns^	1.000 ± 0.039	0.075 ± 0.009	11
Chuxiong	−1.372 ^ns^	1.129 ^ns^	0.955 ± 0.057	0.035 ± 0.012	10
Dali	1.246 ^ns^	−0.796 ^ns^	1.000 ± 0.030	0.073 ± 0.007	13
Dehong	0 ^ns^	4.369 ^ns^	1.000 ± 0.500	0.092 ± 0.046	2
Honghe	−1.376 ^ns^	−6.534 **	0.992 ± 0.025	0.010 ± 0.001	15
Kunming	−0.465 ^ns^	−7.111 *	1.000 ± 0.012	0.037 ± 0.006	24
Lijiang	−0.465 ^ns^	−5.605 *	1.000 ± 0.017	0.029 ± 0.005	19
Lincang	0.070 ^ns^	−3.394 ^ns^	1.000 ± 0.016	0.057 ± 0.005	20
Puer	−0.207 ^ns^	−0.039 ^ns^	1.000 ± 0.126	0.015 ± 0.002	5
Qujing	−0.325 ^ns^	−0.060 ^ns^	0.893 ± 0.111	0.007 ± 0.001	6
Wenshan	0.987 ^ns^	−0.319 ^ns^	0.992 ± 0.025	0.063 ± 0.006	15
Yuxi	−2.017 **	−24.202 ***	0.999 ± 0.004	0.020 ± 0.005	51
Zhaotong	0 ^ns^	2.956 ^ns^	1.000 ± 0.272	0.068 ± 0.029	3

ns: Not significant; *: *p* < 0.05; **: *p* < 0.01; ***: *p* < 0.001.

**Table 6:** Selection pressure analysis of *CP* gene in 202 ChiVMV Yunnan isolates.

Population	Non-Synonymous Mutation (*d*N)	Non-Synonymous Mutation (*d*S)	*d*N/*d*S
All	0.011	0.210	0.051
Baoshan	0.019	0.363	0.053
Chuxiong	0.010	0.152	0.063
Dali	0.018	0.358	0.050
Dehong	0.039	0.342	0.113
Honghe	0.004	0.033	0.107
Kunming	0.007	0.169	0.039
Lijiang	0.007	0.119	0.057
Lincang	0.014	0.251	0.057
Puer	0.006	0.046	0.129
Qujing	0.002	0.027	0.058
Wenshan	0.015	0.292	0.052
Yuxi	0.005	0.085	0.061
Zhaotong	0.020	0.309	0.063

## Data Availability

The raw data supporting the conclusions of this article will be made available by the authors upon request.

## Supplementary Materials

The following supporting information can be downloaded at https://www.mdpi.com/article/10.3390/v18080851/s1, Figure S1: Amino acid sequences consistency of the CP of ChiVMV Yunnan isolates. The upper curve represents the Shannon entropy of amino acids calculated along the CP protein alignment position, used to demonstrate the degree of variation at different amino acid sites. The central matrix displays the amino acid differences of each CP protein sequence relative to the consensus sequence, with light gray indicating consistency with the consensus sequence and colored short lines indicating different types of amino acid substitutions or deletions. The substitution types are classified according to the physicochemical properties of amino acids as Hydrophobic, Polar, Positive, Negative, G/P, and Stop/X/Gap. The left band represents the amino acid consistency between each sequence and the consensus protein sequence. The bottom track represents the distribution of variant amino acid sites in the 287aa CP protein. The lower right triangle heatmap represents the pairwise amino acid consistency between samples, with darker colors indicating higher protein sequence similarity.; Figure S2: Comparative genetic structure of the *CP* sequences of ChiVMV Yunnan isolates across different sampling years. (A) Classical MDS analysis based on the pairwise p-distance matrix of *CP* genes, with different colors representing different sampling years. (B) Circular topology display of *CP* gene maximum likelihood tree, with the outer circle color representing the sampling year. (C) The genetic distance distribution from each sampling year sample to the corresponding year center point is represented by violin plots and box plots, indicating the distribution range and median. (D) The PERMANOVA permutation test results based on sampling year grouping, with a bar chart representing the pseudo-F distribution obtained by permutation and an orange vertical line representing the observed pseudo-F values; The explanatory power of the sampling year on the genetic structure of *CP* is low but reaches a significant level (R^2^=0.038, P=0.0123); Table S1: Sample information of ChiVMV isolates obtained from tobacco-planting regions in Yunnan Province (XLS file); Table S2: ChiVMV isolates from other regions and other hosts used for constructing phylogenetic tree; Table S3: Haplotype of the *CP* gene in ChiVMV Yunnan isolates; Table S4. Information on ChiVMV Yunnan isolates for constructing a phylogenetic tree (XLS file).
