# Supplementary material for: Genetic Diversity and Phylogeny of Chilli Veinal Mottle Virus in Yunnan Tobacco-Planting Regions Based on the CP Gene
Source: Viruses. 2026 Aug 4;18(8):851. doi: 10.3390/v18080851 (PMC13517847; doi:10.3390/v18080851)
Supplement: Supplementary file 1 [file viruses-18-00851-s001.zip › Figure S1. Amino acid sequences consistency of the CP of ChiVMV Yunnan isolates.pdf]

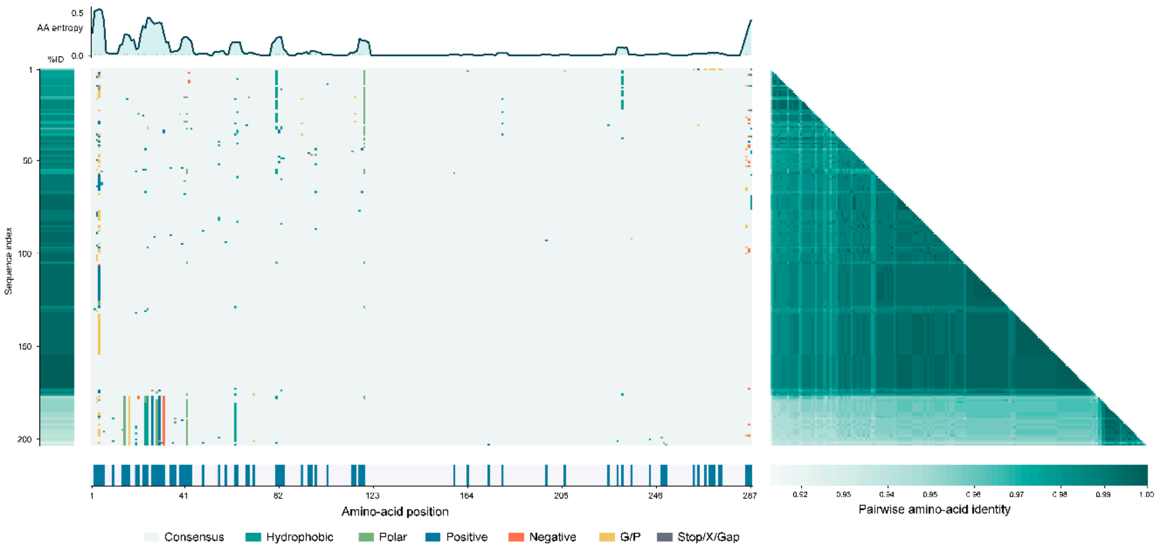

Figure S1. Amino acid sequences consistency of the CP of ChiVMV Yunnan isolates. The upper curve represents the Shannon entropy of amino acids calculated along the CP protein alignment position, used to demonstrate the degree of variation at different amino acid sites. The central matrix displays the amino acid differences of each CP protein sequence relative to the consensus sequence, with light gray indicating consistency with the consensus sequence and colored short lines indicating different types of amino acid substitutions or deletions. The substitution types are classified according to the physicochemical properties of amino acids as Hydrophobic, Polar, Positive, Negative, G/P, and Stop/X/Gap. The left band represents the amino acid consistency between each sequence and the consensus protein sequence. The bottom track represents the distribution of variant amino acid sites in the 287aa CP protein. The lower right triangle heatmap represents the pairwise amino acid consistency between samples, with darker colors indicating higher protein sequence similarity.
