# Supplementary material for: Genetic Diversity and Phylogeny of Chilli Veinal Mottle Virus in Yunnan Tobacco-Planting Regions Based on the CP Gene
Source: Viruses. 2026 Aug 4;18(8):851. doi: 10.3390/v18080851 (PMC13517847; doi:10.3390/v18080851)
Supplement: Supplementary file 1 [file viruses-18-00851-s001.zip › Figure S2. Comparative genetic structure of the CP sequences of ChiVMV Yunnan isolates across different sampling years.pdf]

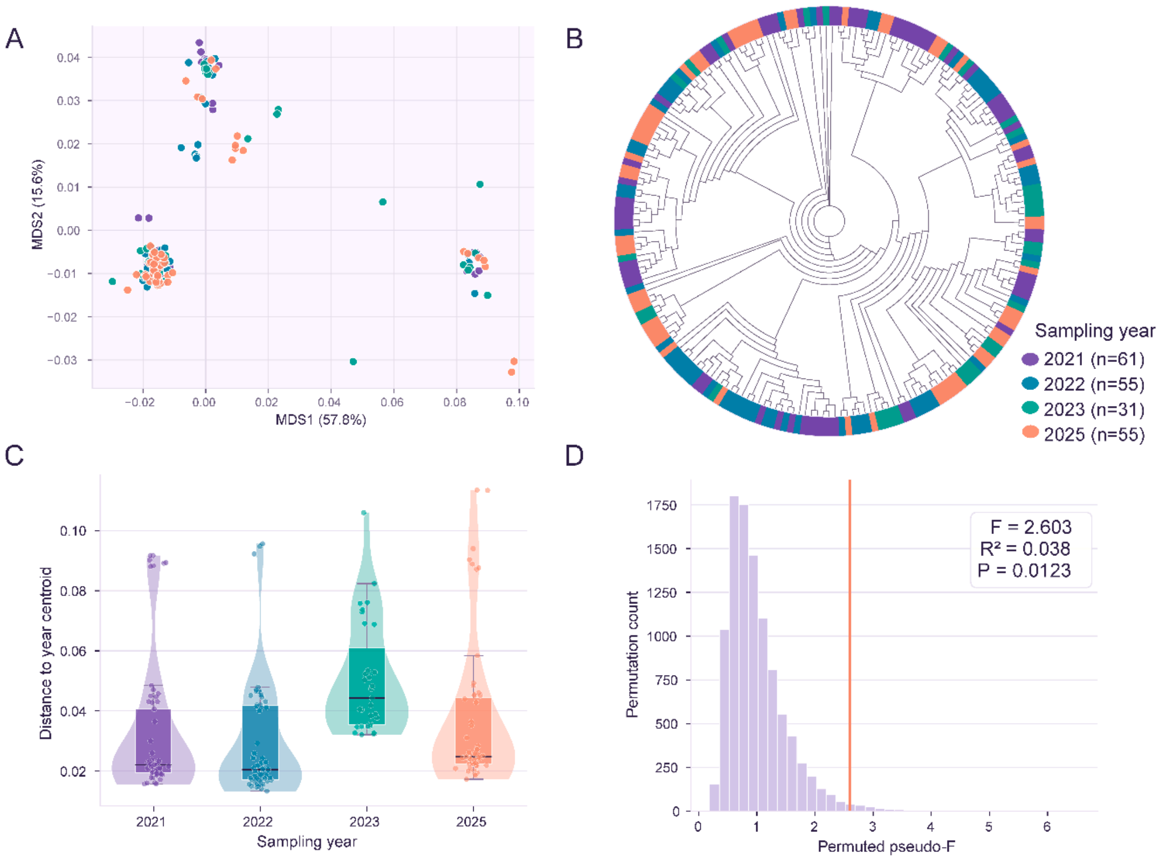

Figure S2. Comparative genetic structure of the *CP* sequences of ChiVMV Yunnan isolates across different sampling years. **A.** Classical MDS analysis based on the pairwise p-distance matrix of *CP* genes, with different colors representing different sampling years. **B.** Circular topology display of *CP* gene maximum likelihood tree, with the outer circle color representing the sampling year. **C.** The genetic distance distribution from each sampling year sample to the corresponding year center point is represented by violin plots and box plots, indicating the distribution range and median. **D.** The PERMANOVA permutation test results based on sampling year grouping, with a bar chart representing the pseudo-F distribution obtained by permutation and an orange vertical line representing the observed pseudo-F values; The explanatory power of the sampling year on the genetic structure of *CP* is low but reaches a significant level (R^2=0.038, P=0.0123).
