# Supplementary material for: Genetic Diversity and Phylogeny of Chilli Veinal Mottle Virus in Yunnan Tobacco-Planting Regions Based on the CP Gene
Source: Viruses. 2026 Aug 4;18(8):851. doi: 10.3390/v18080851 (PMC13517847; doi:10.3390/v18080851)
Supplement: Supplementary file 1 [file viruses-18-00851-s001.zip › Table S2. ChiVMV isolates from other regions and other hosts used for constructing phylogenetic tree.pdf]

Table S2. ChiVMV isolates from other regions and other hosts used for constructing phylogenetic tree

NO.	Accession No.	Origin	Host
1	PQ351398.1	Yunnan, China	*Colocasia esculenta*
2	PQ351399.1	Yunnan, China	*Cucurbita moschata*
3	OM829893.1	Yunnan, China	*Solanum muricatum*
4	OP748367.1	Yunnan, China	*Solanum melongena*
5	PQ368308.1	Yunnan, China	*Bidens pilosa*
6	ON838425.1	Yunnan, China	*Solanum nigrum*
7	PQ466185.1	Yunnan, China	*Datura innoxia*
8	MT974520.1	Yunnan, China	*Capsicum annuum*
9	MG674071.1	Liaoning, China	*Solanum nigrum*
10	KU987835.1	Guangdong, China	*Capsicum annuum*
11	GQ981316.1	Hainan, China	*Capsicum annuum*
12	MT782116.1	Guangxi, China	*Capsicum annuum*
13	OP378160.1	Guizhou, China	*Nicotiana tabacum*
14	PP582383.1	Jiangsu, China	*Nicotiana tabacum*
15	MK405594.1	Sichuan, China	*Nicotiana tabacum*
16	KC711055.1	Sichuan, China	*Capsicum annuum*
17	PX687011.1	Sichuan, China	*Capsicum annuum*
18	KR296797.1	Hunan, China	*Capsicum annuum.*
19	OM746183.1	Saudi Arabia	*Solanum lycopersicum*
20	MK165661.1	India	*Solanum lycopersicum*
21	AB703255.1	Indonesia	*Capsicum annuum*
22	U72193.1	Thailand	*Capsicum annuum*
23	KX236451.1	Pakistan	*Capsicum annuum*
24	KX889918.1	Italy	*Capsicum annuum*
25	DQ925440.1	Vietnam	*Capsicum annuum*
26	OM108478.1	America	*Basella alba*
