# Supplementary material for: Genetic Diversity and Phylogeny of Chilli Veinal Mottle Virus in Yunnan Tobacco-Planting Regions Based on the CP Gene
Source: Viruses. 2026 Aug 4;18(8):851. doi: 10.3390/v18080851 (PMC13517847; doi:10.3390/v18080851)
Supplement: Supplementary file 1 [file viruses-18-00851-s001.zip › Table S3. Haplotype of the CP gene in ChiVMV Yunnan isolates.pdf]

Table S3. Haplotype of the *CP* gene in ChiVMV Yunnan isolates

Haplotype	Number	Name	Geographical origin
Hap_1	1	21CNTB3-11	Baoshan
Hap_2	1	22BSLJ4-1	Baoshan
Hap_3	1	23LJ1-2	Baoshan
Hap_4	1	23LJ4-2	Baoshan
Hap_5	1	25BSLL-11	Baoshan
Hap_6	1	25BSLL-19	Baoshan
Hap_7	1	25BSLL-3	Baoshan
Hap_8	1	25BSLLLc-1	Baoshan
Hap_9	1	25BSTC-14	Baoshan
Hap_10	1	25BSTC-20	Baoshan
Hap_11	1	25BSTC-21	Baoshan
Hap_12	1	23C23-1	Yuxi
Hap_13	1	KC711055.1	Chengdu
Hap_14	3	21CXNH-3、21CXNH-4、21NHSC3-8	Chuxiong
Hap_15	1	21CXWDYJG11-2	Chuxiong
Hap_16	1	21CXYRMH8-5	Chuxiong
Hap_17	2	21NHSC3-14、21MLMY2-10	Chuxiong、Honghe
Hap_18	1	21NHSC3-4	Chuxiong
Hap_19	1	21NHSC3-9	Chuxiong
Hap_20	1	21YAGJ6-7	Chuxiong
Hap_21	1	25CXWD-3	Chuxiong
Hap_22	1	25CXYR-14	Chuxiong
Hap_23	1	25CXYR-15	Chuxiong
Hap_24	1	21DLDEG-1	Dali
Hap_25	1	21DLDEG-2	Dali
Hap_26	1	21DLDH-10	Dali

Hap_27	1	21DLDH-4	Dali
Hap_28	1	21DLDH-6	Dali
Hap_29	1	21DLDH-9	Dali
Hap_30	1	21DLGS-33	Dali
Hap_31	1	23NJG-2	Dali
Hap_32	1	25DLDL-3	Dali
Hap_33	1	25DLDL-9	Dali
Hap_34	1	25DLYL-13	Dali
Hap_35	1	25DLYP-14	Dali
Hap_36	1	25DLYP-7	Dali
Hap_37	1	23LC-1	Dehong
Hap_38	1	Nt23RL1-1	Dehong
Hap_39	2	21JSLA4-2、21CJ-69	Honghe、Yuxi
Hap_40	1	21JSLA4-6	Honghe
Hap_41	1	21ML-30	Honghe
Hap_42	1	21MLMY2-1	Honghe
Hap_43	2	21MLMY2-11、21MLMY2-13	Honghe
Hap_44	1	21MLMY2-7	Honghe
Hap_45	1	22MLHX-1	Honghe
Hap_46	1	22MLHX-2	Honghe
Hap_47	1	22MLXY-1	Honghe
Hap_48	1	22SPDX-1	Honghe
Hap_49	1	22SPG2-17	Honghe
Hap_50	1	22SPYLG-2	Honghe
Hap_51	1	25HHML-2	Honghe
Hap_52	1	25HHML-4	Honghe
Hap_53	2	21SLG1-1、21ZTCYG-4	Kunming、Zhaotong
Hap_54	1	21SLG1-2	Kunming

Hap_55	1	21SLG4-7	Kunming
Hap_56	1	21SLG4-8	Kunming
Hap_57	1	21SLG4-9	Kunming
Hap_58	1	21SLG5-1	Kunming
Hap_59	1	22KSM1-3	Kunming
Hap_60	1	22KSM1-4	Kunming
Hap_61	1	22KSM3-1	Kunming
Hap_62	1	22KSMG-1	Kunming
Hap_63	1	22SL1-4	Kunming
Hap_64	1	22SL1-6	Kunming
Hap_65	1	22SL1-8	Kunming
Hap_66	1	22SL2-3	Kunming
Hap_67	1	22SL2-5	Kunming
Hap_68	1	22SL2-7	Kunming
Hap_69	1	22SL3-3	Kunming
Hap_70	1	22XD1-5	Kunming
Hap_71	1	22XD2-1	Kunming
Hap_72	1	22XD3-1	Kunming
Hap_73	1	25KMSM-6	Kunming
Hap_74	1	25KMSM-7	Kunming
Hap_75	1	25KMXD-2	Kunming
Hap_76	1	25KMXD-5	Kunming
Hap_77	1	22HP1-2	Lijiang
Hap_78	1	22HP2-1	Lijiang
Hap_79	1	22HP3-1	Lijiang
Hap_80	1	22HP3-5	Lijiang
Hap_81	1	22ZX1-2	Lijiang
Hap_82	1	22ZX1-5	Lijiang

Hap_83	1	22ZX2-1	Lijiang
Hap_84	1	22ZX2-6	Lijiang
Hap_85	1	22ZXG2-2	Lijiang
Hap_86	1	25HPWLCCY-1	Lijiang
Hap_87	1	25HPWLYY121-1	Lijiang
Hap_88	1	25HPWLYY301-1	Lijiang
Hap_89	1	25HPXZCCY-1	Lijiang
Hap_90	1	25HPXZSn-2	Lijiang
Hap_91	1	25HPXZSn-4	Lijiang
Hap_92	1	25HPXZSn-5	Lijiang
Hap_93	1	25LJHP-11	Lijiang
Hap_94	1	25LJHP-9	Lijiang
Hap_95	1	Nt22ZX1-1	Lijiang
Hap_96	1	22LCSJ-3	Lincang
Hap_97	1	22LCSJ-4	Lincang
Hap_98	1	23GM4-5	Lincang
Hap_99	1	23GM5-14	Lincang
Hap_100	1	23GM5-16	Lincang
Hap_101	1	23LX2-13	Lincang
Hap_102	1	23LX3-6	Lincang
Hap_103	1	23LX3-7	Lincang
Hap_104	1	23SJ2-11	Lincang
Hap_105	1	23SJ2-4	Lincang
Hap_106	1	23SJ2-5	Lincang
Hap_107	1	23SJ3-7	Lincang
Hap_108	1	25LCLX-10	Lincang
Hap_109	1	25LCLX-11	Lincang
Hap_110	1	25LCLX-23	Lincang

Hap_111	1	25LCLX-26	Lincang
Hap_112	1	25LCLX-32	Lincang
Hap_113	1	25LCLX-4	Lincang
Hap_114	1	25LCYD-1	Lincang
Hap_115	1	25LCYD-2	Lincang
Hap_116	1	22GC1-2	Puer
Hap_117	1	22GC3-1	Puer
Hap_118	1	22JD1-2	Puer
Hap_119	1	22JD1-5	Puer
Hap_120	1	22ZY7-1	Puer
Hap_121	1	21ZYDP-19	Qujing
Hap_122	3	21ZYDP-31、21ZYDP-41、21ZYDP-56	Qujing
Hap_123	1	23LP1-3	Qujing
Hap_124	1	23LP3-4	Qujing
Hap_125	1	25QJZY-1	Qujing
Hap_126	1	25QJZY-10	Qujing
Hap_127	1	21YSJN3-11	Wenshan
Hap_128	1	21YSJN3-4	Wenshan
Hap_129	2	22GNLY-1、22GNLY-2	Wenshan
Hap_130	1	22YSWM1-1	Wenshan
Hap_131	1	22YSWM1-2	Wenshan
Hap_132	1	23QB1-1	Wenshan
Hap_133	1	23QB1-3	Wenshan
Hap_134	1	23QB1-4	Wenshan
Hap_135	1	23QBSP1-2	Wenshan
Hap_136	1	23WSQB2-1	Wenshan
Hap_137	1	23WSQB2-2	Wenshan
Hap_138	1	23WSQBG3-1	Wenshan

Hap_139	1	23WSQBG3-3	Wenshan
Hap_140	1	25WSQB-1	Wenshan
Hap_141	1	25WSQB-5	Wenshan
Hap_142	1	21CJ-13	Yuxi
Hap_143	1	21CJ-14	Yuxi
Hap_144	1	21CJ-15	Yuxi
Hap_145	1	21CJ-16	Yuxi
Hap_146	1	21CJ-49	Yuxi
Hap_147	1	21CJ-50	Yuxi
Hap_148	1	21CJ-58	Yuxi
Hap_149	1	21HN-19	Yuxi
Hap_150	1	21HN-24	Yuxi
Hap_151	1	21HNG-12	Yuxi
Hap_152	1	21HNG-8	Yuxi
Hap_153	1	21XPG-31	Yuxi
Hap_154	1	21YXHNXS-2	Yuxi
Hap_155	1	21YXHNXT-3	Yuxi
Hap_156	1	21YXTH-18	Yuxi
Hap_157	1	21YXTH-19	Yuxi
Hap_158	1	21YXTH1-33	Yuxi
Hap_159	1	21YXTH1-35	Yuxi
Hap_160	1	21YXTH1-43	Yuxi
Hap_161	1	21YXTH4-79	Yuxi
Hap_162	1	21YXTHG2-1	Yuxi
Hap_163	1	21YXTHG2-2	Yuxi
Hap_164	1	22CJ1-2	Yuxi
Hap_165	1	22CJ3-1	Yuxi
Hap_166	1	22CJ3-2	Yuxi

Hap_167	1	22JCAH-1	Yuxi
Hap_168	1	22JCAH-2	Yuxi
Hap_169	1	22JCGD-1	Yuxi
Hap_170	1	22JCGDG-1	Yuxi
Hap_171	1	22JCJC-1	Yuxi
Hap_172	1	22JX-1	Yuxi
Hap_173	1	22JX-2	Yuxi
Hap_174	1	22JX-3	Yuxi
Hap_175	1	22TH01	Yuxi
Hap_176	1	22YJ-2	Yuxi
Hap_177	1	23CJ1	Yuxi
Hap_178	1	23DZ1	Yuxi
Hap_179	2	23XJ1-1、23XJ1-2	Yuxi
Hap_180	1	25YXCJ-1	Yuxi
Hap_181	1	25YXCJ-3	Yuxi
Hap_182	1	25YXCJ-7	Yuxi
Hap_183	1	25YXCJBp-2	Yuxi
Hap_184	1	25YXES-3	Yuxi
Hap_185	1	25YXES-4	Yuxi
Hap_186	1	25YXES-5	Yuxi
Hap_187	1	25YXESCa-1	Yuxi
Hap_188	1	25YXHN-4	Yuxi
Hap_189	1	25YXHN-7	Yuxi
Hap_190	1	25YXXP-3	Yuxi
Hap_191	1	25YXXP-4	Yuxi
Hap_192	1	23ZT5-6	Zhaotong
Hap_193	1	23ZT5-7	Zhaotong
